# Intra-cluster correlation coefficients in primary care patients with type 2 diabetes and hypertension

**DOI:** 10.1186/s13063-020-04349-4

**Published:** 2020-06-16

**Authors:** Yi Lin Lee, Yvonne Mei Fong Lim, Kian Boon Law, Sheamini Sivasampu

**Affiliations:** 1 Centre for Clinical Trial, Institute for Clinical Research, Ampang Hospital, Ministry of Health, Jalan Mewah Utara, Pandan Mewah, 68000 Ampang, Selangor Malaysia; 2 Centre for Clinical Outcome Research, Institute for Clinical Research, National Institute of Health, Ministry of Health, Kompleks Institut Kesihatan Negara (NIH), No. 1, Jalan Setia Murni U13/52, Seksyen U13, Setia Alam, 40170 Shah Alam, Selangor Malaysia

**Keywords:** Intra-cluster correlation, Type 2 diabetes, Hypertension, Primary care, Sample size

## Abstract

**Introduction:**

There are few sources of published data on intra-cluster correlation coefficients (ICCs) amongst patients with type 2 diabetes (T2D) and/or hypertension in primary care, particularly in low- and middle-income countries. ICC values are necessary for determining the sample sizes of cluster randomized trials. Hence, we aim to report the ICC values for a range of measures from a cluster-based interventional study conducted in Malaysia.

**Method:**

Baseline data from a large study entitled Evaluation of Enhanced Primary Health Care interventions in public health clinics (EnPHC-EVA: Facility) were used in this analysis. Data from 40 public primary care clinics were collected through retrospective chart reviews and a patient exit survey. We calculated the ICCs for processes of care, clinical outcomes and patient experiences in patients with T2D and/or hypertension using the analysis of variance approach.

**Results:**

Patient experience had the highest ICC values compared to processes of care and clinical outcomes. The ICC values ranged from 0.01 to 0.48 for processes of care. Generally, the ICC values for processes of care for patients with hypertension only are higher than those for T2D patients, with or without hypertension. However, both groups of patients have similar ICCs for antihypertensive medications use. In addition, similar ICC values were observed for clinical outcomes, ranging from 0.01 to 0.09. For patient experience, the ICCs were between 0.03 (proportion of patients who are willing to recommend the clinic to their friends and family) and 0.25 (for Patient Assessment of Chronic Illness Care item 9, Given a copy of my treatment plan).

**Conclusion:**

The reported ICCs and their respective 95% confidence intervals for T2D and hypertension will be useful for estimating sample sizes and improving efficiency of cluster trials conducted in the primary care setting, particularly for low- and middle-income countries.

## Introduction

Cluster randomized controlled trials (CRCTs) are increasingly being used to study the impact of practice intervention in primary care settings [1, 2]. It is known that the application of cluster randomization is less demanding to clinical practice and more administratively convenient [3]. In CRCTs, subjects are not allocated to intervention independently, but as a group within a cluster. Examples of clusters include communities, schools, clinics and hospitals [4]. Compared to individually randomized trials, CRCTs are not only more complex in design and analysis, but also require more participants to reach equivalent statistical power, as observations in the same cluster tend to be more “alike” [5, 6]. The condition whereby the effective sample size is less than the total number of participants is referred to as the clustering effect in CRCTs.

The clustering effect can be determined by calculating the intra-cluster correlation coefficient (ICC), ρ = $\frac{b}{b+w}$ , based on measured outcomes, where *b* is the between-cluster variance and *w* is the within-cluster variance [5]. Therefore, the ICC can range from 0 to 1, depending on how varied the measured outcomes would be within a cluster. The sample size of CRCTs often needs to be inflated by the design effect, DE = 1 + (*n* – 1) ρ, where *n* is the average number of individuals per cluster. For instance, when there is no clustering effect on a measured outcome, the calculated ICC would be near to zero and the estimated DE may be very close to 1. A small DE indicates that the clustering effect does not inflate the sample size [7, 8].

There have been repeated calls to publish ICCs for future cluster-based interventional studies [7, 9]. Several researchers have published ICCs from large primary care trials conducted in high-income countries [2, 7, 10, 11]. In this paper, we report information on ICCs from a large study conducted at the public primary care clinics in Malaysia, which is an upper middle-income country. Based on the measured variables, we report the ICCs of processes of care, intermediate clinical outcomes and patients’ experience of self-management support amongst patients with type 2 diabetes (T2D) with or without hypertension and patients with hypertension only clustered within the selected primary care clinics. The objective of reporting ICCs is to aid sample size estimation for future CRCTs conducted at the primary healthcare level in low- and middle-income countries, as countries in this income category are more similar in the structure and resources of their healthcare systems compared to high-income countries.

## Method

This study used baseline data from a larger study called the Evaluation of Enhanced Primary Health Care (EnPHC) interventions in public health clinics (EnPHC-EVA: Facility). We reported ICC estimates for a range of process and outcome measures for T2D and hypertension based on an ICC reporting framework recommended by a group of researchers and statisticians with experience in CRCTs [12].

### The EnPHC interventions and evaluation study

EnPHC-EVA: Facility was a quasi-experimental controlled study which aimed to assess the effectiveness of an intervention package, known as the EnPHC package, amongst patients with T2D and hypertension in 40 public primary care clinics in Malaysia. Twenty clinics received the interventions, whilst another 20 served as controls. Eligible patients were Malaysians aged 30 years and older who were diagnosed with T2D and/or hypertension. Patients who were pregnant were excluded. Data were collected via retrospective chart review, patient exit survey, healthcare provider survey and an intervention checklist. The effect of the interventions was then analysed using two quasi-experimental analytical methods: the interrupted time series and difference-in-differences approaches. The EnPHC-EVA: Facility study has been completed, and a detailed protocol is currently under journal review.

The clinics involved were public primary healthcare clinics located in the central and southern states of Malaysia, namely Selangor and Johor. These clinics were selected by the study implementers based on the budget and capacity to implement the EnPHC package. In the public primary care setting, there are seven clinic types, type 1 to type 7 [13]. These clinic types are classified by total daily attendances; type 1 is the largest with more than 800 patient visits a day, and type 7 has less than 50 visits daily. The clinics included within this sample were between types 2 to 4, which represented about 45% of the public primary clinics in Malaysia based on an unpublished Family Health Development Division report in 2019. The selection criteria for the clinics were at least two medical doctors and 300–800 patient attendances daily. The clinics were then matched in pairs based on the number of medical doctors, number of family medicine specialists, geographical location (urban or rural), annual patient attendance and availability of electronic medical records before being randomly allocated to intervention and control groups.

### The EnPHC package included three aspects:

1. *Community engagement*. People in the community who resided within the catchment area of the intervention clinics were assigned to appropriate health programs according to their respective cardiovascular risks.
2. *Person-centred care bundles*. These included cardiovascular risk stratification, assignment to family health teams and task shifting from doctors to other healthcare professionals to improve the continuity and comprehensiveness of chronic care.
3. *Integrated care network*. Information flow and continuity of care between primary care and other levels of care were improved through standardized referral forms and the role of a care coordinator.

months): (1) November 2016 to June 2017 (one pre-intervention phase), (2) August 2017 to June 2019 (three post-intervention phases). For the present analysis, only baseline data from November 2016 to June 2017 were included. Clinic visits were sampled separately according to patients’ diagnosis of T2D with or without hypertension or hypertension only, because patient registration and appointments were categorized by these groups in the public clinics. Visits were also stratified by the month of their visits, because the analysis on intervention effectiveness also assessed changes in processes of care and outcomes with time. Then, a sampling frame for the clinic visits was created accordingly from the patients’ register and samples were selected by systematic random sampling, with the random start number generated using Microsoft Excel. Systematic random sampling was used instead of simple random sampling, because it was more straightforward and practical to implement, given the large number of samples by time points that needed to be drawn [14].

The measurements of interest in this study were processes of care, intermediate clinical outcomes and patients’ experience. The processes of care and intermediate clinical outcomes were collected from clinic visit records via retrospective chart review using a standardized data extraction form. Patients’ experience was assessed using a questionnaire administered by trained researchers to the patients via face-to-face interview.

We reported ICC values for processes of care and therapeutic targets according to the standard of care for T2D and hypertension [15, 16]. Examples of the processes of care evaluated are at least one HbA1c measurement within 3 months, a foot examination done within 3 months and a lipid measurement within the past 12 months, whilst the intermediate clinical outcomes include the percentage of patients who achieve HbA1c values of ≤8% and the percentage of patients who achieve target blood pressure values of ≤135/75 mmHg. We also reported the mean values of these intermediate outcomes, because some researchers may want to calculate their study sample sizes based on mean differences.

Patients’ experience on self-management support was assessed using a short version of the original Patient Assessment of Chronic Illness Care (PACIC) instrument [17]. The questionnaire contained 3 subscales and 11 items [18], as compared to the original PACIC (5 subscales and 20 items). In order to prevent respondent fatigue and after taking into account the low health literacy level amongst the study population, a consensus was reached by an expert group to only adapt the first 11 items, which are from the first 3 subscales of PACIC (patient activation, delivery system design/decision support and goal-setting/tailoring). Each item can be scored by choosing from the options of “None of the time (1)”, “A little of the time (2)”, “Some of the time (3)”, “Most of the time (4)” and “Always (5)”. “None of the time” has a score of 1 and “Always” has a score of 5. The score of each subscale can be calculated by averaging the scores of the items within that subscale [17].

We defined the processes of care and intermediate clinical outcomes as objective measures, whilst patient-reported experience was considered a subjective measure.

Ethical approval was granted by the Medical Research and Ethics Committee, Ministry of Health Malaysia (study registration number NMRR-17-267-34768).

### Data analysis

Analyses were undertaken using R version 3.6.2. Missing data ranged from 0.2% to 27.1%, and complete case analysis was performed. The R scripts are provided in the Appendix.

A standard one-way analysis of variance (ANOVA) was conducted to generate the ICC by using the mean square values. The estimated ICC is given by the formula [19]

$$ \rho =\frac{MS_{between}-{MS}_{within}}{MS_{between}+\left(m-1\right){MS}_{within}} $$

where *MS* indicates the mean square values from ANOVA and *m* is the average cluster size. The 95% confidence interval (CI) was estimated using Smith’s large sample approximation [20, 21], which caters for large and normally distributed data.

For binary variables, we used the ANOVA method with Smith’s large sample approximation [20, 21] in the ICCbin package [20] to generate the ICC and 95% CI. Examples of binary variables were whether a process of care such as foot examination was conducted for a diabetes patient and the proportion of patients achieving target values for intermediate clinical outcomes.

For continuous data such as the PACIC score and mean intermediate clinical outcome values such as for HbA1c, ICCs were estimated using the ICC1.CI function from the psychometric package [22]. This function performs a one-way ANOVA fixed effects model for continuous data. In this model, the differences between the clusters, which are the fixed or discrete effects, were estimated [23].

The analyses were conducted separately for patients with T2D (with or without hypertension) and patients with hypertension only, because the processes of care and therapeutic targets for patients with diabetes are different from those who only have hypertension. We reported unadjusted ICC values, because unadjusted values were generally recommended for use in sample size calculation [24]. However, researchers should be aware that published ICCs are estimates only, and they are advised to make statistical adjustment during analysis [24].

## Results

A total of 6722 subjects with T2D (with or without hypertension) and 5014 subjects with hypertension only were included in this study for the reporting of results on processes of care and intermediate clinical outcomes. For the patient exit survey, 956 patients with T2D and/or hypertension were interviewed. The baseline characteristics of the study population are described in Table 1. The mean age of the study population was 60 years old. The patients were predominantly female (62%) and of Malay (69%) ethnicity. The majority of the patients (82%) were overweight or obese, and about 42% of them had hyperlipidaemia. The average cluster size ranged from 141 to 168 for T2D patients with or without hypertension and from 94 to 126 for hypertension-only groups.

**Table 1:** Baseline characteristics of study population

Characteristics	Mean (SD) or ***n*** (%)
**Study population for process of care and clinical outcomes (*n*** **= 11,736)**	
Age, years	60.3 (11.3)
Sex = female	7323 (62.4)
Body mass index	27.9 (5.8)
Body mass index, kg/m^2^ [25]	
< 18.5 (underweight)	225 (1.9)
18.5–22.9 (normal)	1317 (11.2)
23–27.4 (overweight)	3001 (25.6)
> 27.4 (obese)	4250 (36.2)
Ethnicity	
Malay	8071 (68.8)
Chinese	2456 (20.9)
Indian	1112 (9.5)
Other	95 (0.8)
Morbidity/risk factor	
T2D	6722 (57.3)
Hypertension	10,271 (87.5)
Hyperlipidaemia	4951 (42.2)
Duration of illness, years	
T2D	6.5 (4.3)
Hypertension	7.5 (5.1)
Hyperlipidaemia	5.0 (3.3)
Target organ damage = Yes	2591 (22.1)
**Study population for patient experience (*n*** **= 956)**	
Age, years	59.7 (10.9)
Sex = female	576 (60.2)
Ethnicity	
Malay	664 (69.5)
Chinese	200 (20.9)
Indian	88 (9.2)
Other	4 (0.4)
Educational level	
No formal/primary/lower secondary	659 (68.9)
Upper secondary	239 (25.0)
Tertiary	58 (6.1)
Morbidity/risk factor	
T2D	623 (65.2)
Hypertension	808 (84.5)
Hyperlipidaemia	431 (45.1)
Duration of illness, years	
T2D	8.2 (7.0)
Hypertension	8.4 (7.9)
Hyperlipidaemia	5.2 (4.5)
**Clinic characteristics *n***=**40**	
Geographical location	
Rural	18 (45.0)
Urban	22 (55.0)
Daily attendances in 2016	282 (147)

*SD* standard deviation, *T2D* type 2 diabetes

**Fig. 1:**
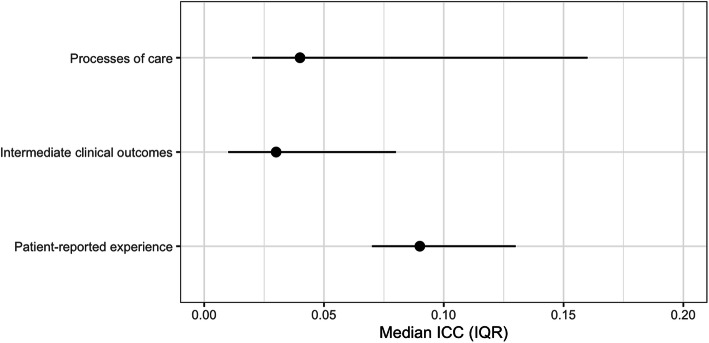
Median intra-cluster correlation coefficients (ICCs) by processes of care, intermediate clinical outcomes and patient-reported experience, with their respective interquartile ranges (IQRs)

ICCs for processes of care, intermediate clinical outcomes and patient experience are presented in Tables 2, 3 and 4 respectively. Between the three categories of measures, the highest ICC was observed for patient-reported experience with a median ICC of 0.09 (interquartile range [IQR] 0.07, 0.13) followed by processes of care with a median of 0.04 (IQR 0.02, 0.16) and intermediate clinical outcomes with a median of 0.03 (IQR 0.01, 0.08) (see Fig. 1).

**Table 2:** Intra-cluster correlation coefficients for processes of care

Processes of care	Number, ***N***	Average cluster size	***n*** (%)	ICC	95% CI
**T2D PATIENTS WITH OR WITHOUT HYPERTENSION (*N*** **= 6722)**					
**Test/assessment done within a specific interval**					
**Test/assessment done on visit day**					
FBG	6722	168	3053 (45.4)	0.18	0.11, 0.25
RBG	6722	168	2685 (39.9)	0.14	0.08, 0.19
Blood pressure measurement	6707	168	6574 (97.8)	0.03	0.01, 0.05
**At least 1 test/assessment within 3 months**					
HbA1c	6722	168	2450 (36.4)	0.09	0.05, 0.13
FBG	6722	168	3797 (56.5)	0.16	0.09, 0.22
Foot examination (pulse, neuropathy, ulcer)	6722	168	1837 (27.3)	0.17	0.10, 0.23
**At least 1 test/assessment within 6 months**					
Body mass index	6696	167	2699 (40.2)	0.48	0.37, 0.59
**At least 1 test/assessment within 12 months**					
Lipid profile					
TC	6722	168	5376 (80.0)	0.03	0.01, 0.04
LDL-C	6722	168	4624 (68.8)	0.16	0.10, 0.22
HDL-C	6722	168	4614 (68.6)	0.16	0.10, 0.23
Triglycerides	6722	168	5300 (78.8)	0.02	0.01, 0.04
Complete lipid profile (TC, LDL-C, HDL-C, triglycerides)	6722	168	4533 (67.4)	0.16	0.10, 0.22
Serum creatinine	6722	168	5508 (81.9)	0.03	0.01, 0.04
Urine albumin	6722	168	3897 (58.0)	0.20	0.13, 0.28
Liver function	6722	168	3644 (54.2)	0.37	0.26, 0.47
Visual acuity examination	6722	168	3336 (49.6)	0.21	0.13, 0.28
Fundus examination	6722	168	2344 (34.9)	0.13	0.08, 0.18
Electrocardiography	6722	168	3248 (48.3)	0.22	0.14, 0.29
**Medication prescribed**					
**Glucose-lowering drugs**					
Biguanide (Metformin)	6722	168	5686 (84.6)	0.01	0.01, 0.02
Sulphonylurea	6722	168	3456 (51.4)	0.02	0.01, 0.03
Insulin	6722	168	1977 (29.4)	0.02	0.01, 0.03
Alpha-glucosidase inhibitor (acarbose)	6722	168	102 (1.5)	0.03	0.02, 0.05
**Antihypertensive drugs**					
ACEI/ARB	6722	168	4242 (63.1)	0.04	0.02, 0.05
Calcium channel blocker	6722	168	4056 (60.3)	0.02	0.01, 0.03
Beta-blocker	6722	168	1717 (25.5)	0.03	0.01, 0.04
Diuretic	6722	168	1535 (22.8)	0.03	0.01, 0.05
Alpha-blocker	6722	168	380 (5.7)	0.02	0.01, 0.03
**Lipid-lowering drugs**					
HMG-CoA reductase inhibitor (statin)	6722	168	5250 (78.1)	0.04	0.02, 0.06
Fibrate	6722	168	164 (2.4)	0.03	0.01, 0.04
**PATIENTS WITH HYPERTENSION (*N*** **= 5014)**					
**Test/assessment done within a specific interval**					
**Test/assessment done on visit day**					
Blood pressure measurement	4985	125	4916 (98.6)	0.05	0.03, 0.08
**At least 1 test/assessment done within 12 months**					
Blood glucose tests (FBG/RBG/HbA1c)	5014	126	3590 (71.6)	0.09	0.05, 0.13
Serum creatinine	5014	126	4005 (79.9)	0.06	0.03, 0.09
Urine albumin	5014	126	2087 (41.6)	0.23	0.15, 0.32
Lipid profile					
TC	5014	126	3919 (78.2)	0.05	0.03, 0.08
LDL-C	5014	126	2954 (58.9)	0.22	0.14, 0.30
HDL-C	5014	126	2969 (59.2)	0.21	0.13, 0.29
Triglycerides	5014	126	3894 (77.7)	0.05	0.03, 0.08
Complete lipid profile (TC, LDL-C, HDL-C, triglycerides)	5014	126	2920 (58.2)	0.21	0.13, 0.29
Electrocardiography	5014	126	1802 (35.9)	0.28	0.18, 0.37
**Medication prescribed**					
**Antihypertensive drugs**					
Calcium channel blocker	5014	126	3972 (79.2)	0.01	0.002, 0.02
ACEI/ARB	5014	126	2210 (44.1)	0.04	0.02, 0.06
Beta-blocker	5014	126	1382 (27.6)	0.02	0.01, 0.04
Diuretic	5014	126	1054 (21.0)	0.02	0.01, 0.03
Alpha-blocker	5014	126	202 (4.0)	0.02	0.01, 0.03
**Lipid-lowering drugs**					
HMG-CoA reductase inhibitor	5014	126	3427 (68.3)	0.04	0.02, 0.06
Fibrate	5014	126	63 (1.3)	0.01	0, 0.01

*ACEI* angiotensin-converting enzyme inhibitor, *ARB* angiotensin II receptor blocker, *CI* confidence interval, *FBG* fasting blood glucose, *HbA1c* glycated haemoglobin, *HDL-C* high-density lipoprotein cholesterol, *HMG-CoA* hydroxymethylglutaryl-coenzyme A, *ICC* intra-cluster correlation coefficient, *LDL-C* low-density lipoprotein cholesterol, *RBG* random blood glucose, *T2D* type 2 diabetes, *TC* total cholesterol

**Table 3:** Intra-cluster correlation coefficients for intermediate clinical outcomes

Intermediate clinical outcomes	Number, ***N***	Average cluster size	Mean (SD) or ***n*** (%)	ICC	95% CI
**T2D PATIENTS WITH OR WITHOUT HYPERTENSION (*N*** **= 6722)**					
HbA1c, %	6208	155	8.3 (2.2)	0.02	0.01, 0.03
HbA1c ≤ 7%	6208	155	2135 (34.4)	0.01	0.002, 0.02
HbA1c ≤ 8%	6208	155	3291 (53.0)	0.01	0.002, 0.02
Systolic BP, mmHg^a^	6712	168	137.8 (17.1)	0.08	0.06, 0.13
Diastolic BP, mmHg^a^	6712	168	77.8 (9.8)	0.08	0.05, 0.12
BP ≤ 130/80 mmHg	6712	168	1936 (28.8)	0.09	0.05, 0.13
BP ≤ 135/75 mmHg	6712	168	1757 (26.2)	0.06	0.03, 0.09
BP ≤ 140/80 mmHg	6712	168	2991 (44.6)	0.08	0.05, 0.12
Serum creatinine (μmol/L)	6455	162	88.6 (69.7)	0.01	0.01, 0.02
TC (mmol/L)	6360	159	5.1 (1.2)	0.03	0.02, 0.06
LDL-C (mmol/L)	5624	141	3.0 (1.1)	0.03	0.02, 0.05
LDL-C ≤ 2.6 mmol/L	5624	141	2191 (39.0)	0.02	0.01, 0.03
HDL-C (mmol/L)	5492	141	1.3 (0.4)	0.03	0.02, 0.05
Triglycerides (mmol/L)	6335	159	1.8 (1.2)	0.01	0.01, 0.03
**PATIENTS WITH HYPERTENSION (*N*** **= 5014)**					
Systolic BP, mmHg^a^	5004	126	137.8 (17.0)	0.08	0.05, 0.13
Diastolic BP, mmHg^a^	5004	126	78.7 (10.9)	0.09	0.06, 0.14
BP < 140/90 mmHg [15]	5004	126	2644 (52.8)	0.05	0.02, 0.07
Serum creatinine (μmol/L)	4738	119	83.2 (64.6)	0.01	0.01, 0.03
TC (mmol/L)	4679	117	5.3 (1.1)	0.03	0.02, 0.06
LDL-C (mmol/L)	3800	95	3.2 (1.0)	0.05	0.03, 0.09
LDL-C ≤ 2.6 mmol/L	3800	95	1082 (28.5)	0.03	0.01, 0.05
HDL-C (mmol/L)	3653	94	1.4 (0.4)	0.03	0.01, 0.05
Triglycerides (mmol/L)	4685	118	1.5 (0.9)	0.01	0.004, 0.02

*BP* blood pressure, *CI* confidence interval, *HbA1c* glycated haemoglobin, *HDL-C* high-density lipoprotein cholesterol, *ICC* intra-cluster correlation coefficient, *LDL-C* low-density lipoprotein cholesterol, *SD* standard deviation, *T2D* type 2 diabetes, *TC* total cholesterol

^a^Systolic BP and diastolic BP values are the average of two readings

**Table 4:** Intra-cluster correlation coefficients for patient experience and willingness to recommend

Patient experience measures	Total	Average cluster size	Mean (SD) or ***n*** (%)	ICC	95% CI
**Patient activation score (1–3)**	**956**	**24**	**2.1 (1.1)**	**0.11**	**0.06, 0.19**
1. Asked for my ideas when we made a treatment plan	956	24	2.1 (1.4)	0.09	0.05, 0.16
2. Given choices about treatment to think about	956	24	1.8 (1.3)	0.09	0.05, 0.16
3. Asked to talk about any problems with my medicines or their effects	956	24	2.3 (1.5)	0.06	0.03, 0.12
**Delivery system design/Practice design score (4–6)**	**956**	**24**	**2.9 (0.9)**	**0.11**	**0.07, 0.20**
4. Given a written list of things I should do to improve my health	956	24	1.6 (1.2)	0.14	0.09, 0.23
5. Satisfied that my care was well organized	956	24	4.2 (1.1)	0.08	0.05, 0.15
6. Shown how what I did to take care of my illness influenced my condition	956	24	2.8 (1.5)	0.06	0.03, 0.12
**Goal setting/Tailoring score (7–11)**	**956**	**24**	**2.1 (0.9)**	**0.13**	**0.08, 0.22**
7. Asked to talk about my goals in caring for my illness	956	24	2.2 (1.5)	0.19	0.12, 0.29
8. Helped to set specific goals to improve my eating or exercise	956	24	2.4 (1.5)	0.09	0.05, 0.16
9. Given a copy of my treatment plan	956	24	2.7 (1.8)	0.25	0.17, 0.36
10. Encouraged to go to a specific group or class to help me cope with my chronic illness	956	24	1.5 (1.0)	0.07	0.03, 0.13
11. Asked questions either directly or on a survey, about my health habits	956	24	1.8 (1.3)	0.11	0.06, 0.18
Proportion of patients who are willing to recommend the clinic to their friends and family	956	24	857 (89.6)	0.03	0, 0.06

*CI* confidence interval, *ICC* intra-cluster correlation coefficient, *SD* standard deviation

For processes of care, the ICC values ranged from 0.01 to 0.48. The highest ICC was for measurement of body mass index (BMI, 0.48) and liver function test within 12 months (0.37) in T2D patients with or without hypertension. Overall, the ICCs for common processes of care for patients with hypertension only were higher than those for T2D patients with or without hypertension. However, both groups of patients had similar ICCs (0.01 to 0.04) for antihypertensive medications use. The results also showed low ICC estimates for medication prescribing across all medication groups, for both T2D patients with or without hypertension and hypertension-only patients.

As for clinical outcomes, two variables had the highest ICCs of 0.09. They were the proportion of T2D patients with or without hypertension who achieved the target blood pressure of ≤130/80 mmHg and the mean diastolic blood pressure of hypertension-only patients. These ICCs for clinical outcomes were mostly similar between both disease groups, ranging from 0.01 to 0.09. Between binary and continuous data types, such as the proportion of T2D patients with or without hypertension who achieved HbA1c ≤ 7% versus the mean value of HbA1c, the ICC values were also similar at around 0.01. This showed that the variations between clusters were largely similar for intermediate clinical outcomes, whether between different patient groups or data types.

For patient experience, the ICC values were between 0.03 (Proportion of patients who are willing to recommend the clinic to their friends and family) and 0.25 (PACIC item 9, Given a copy of my treatment plan). The mean scores for each item in PACIC were at the lower end of the scale, ranging between 1.5 to 2.8, except for item 5 (Satisfied that my care was well organized), which had a mean score of 4.2. Both the “Patient activation” and “Delivery system design/Practice design” subscales had the same ICC values of 0.11. The ”Goal setting/Tailoring” subscale had a slightly higher ICC value (0.13) than the other two subscales. This showed that there were slightly more differences between clinics in engaging patients to set their treatment goal as part of their self-management support initiative. The average cluster size for this group of patients was 23.9 (range 23–29).

## Discussion

To the best of our knowledge, this study is amongst the first to report ICCs for T2D and hypertension processes of care and intermediate clinical outcomes in a middle-income country. An analysis of 31 primary care studies found that the context or setting in which the ICC is derived influences the size of the ICC [26]. Since the majority of research works on ICCs for T2D and hypertension care were from high-income countries [2, 11, 27], it was necessary to report those from our setting to determine if factors such as differences in organizational structure and resource limitations within a healthcare setting are determinants of the magnitude of ICCs. In this study, we found that estimates for process and intermediate outcome indicators were broadly similar to those reported for high-income countries, by Singh et al. and Littenberg et al. for diabetes and hypertension, except for HbA1c and blood pressure readings [7, 11].

We have reported confidence intervals (CIs) together with the ICC estimates, which are useful for investigators who want to perform sensitivity analyses during sample size estimation [26]. Measures of precision are recommended to be included when reporting ICCs, and CIs are the preferred mechanism for providing this information [12]. Bell and McKenzie recommended to examine a range of plausible ICC values and then select conservative options when calculating the sample size for cluster trials [24]. We present these results to expand the existing literature on ICCs in primary care, particularly for low- and middle-income settings.

Our findings were consistent with those reported in the previous literature, where ICCs for processes of care were in general higher than for clinical outcomes, albeit to a lesser extent compared to most studies [7, 11, 28]. A high ICC indicates that the implementation of the processes of care is highly associated with practices. This happens because process measures are largely influenced by health providers’ behaviours, which are likely to be collectively more similar within the same clinic. We found that ICCs for the processes of care for hypertension-only patients are generally higher than those for the same processes of care in T2D patients with or without hypertension. This suggests that the variation in management of hypertension-only patients differs more between clinics compared to that of patients with T2D. On the other hand, clinical outcomes are based on an interaction of differences in biological characteristics, diet, lifestyle practices, attitudes and behaviour towards health; thereby they will exhibit greater variability within clusters, causing the ICC to move towards zero. Amongst all process of care measures, BMI measurement, annual electrocardiography and liver function tests had the highest ICC values. This high variability between clinics reflects differences in provider practice styles, documentation and reminders, availability of resources and time constraints between clinics. Similarly, high ICC values were also reported by Gulliford et al. for the recording of annual weight and urine protein measurement for government primary care services in Trinidad and Tobago [29]. The observed lower median ICC for process measures (0.04) compared to that in the study by Singh and colleagues (0.09) [11] is attributable to the more detailed breakdown of medication categories within this study and the fact that medication prescription has low variation between clinics. This low variation in medication prescription is largely due to the use of a common national drug formulary for public clinics and guideline-adherent practices.

Prescription of glucose-lowering, antihypertensive and lipid-lowering medications had lower ICCs compared to the laboratory tests and assessments, indicating consistency in pharmacological management of patients with diabetes and hypertension across clinics. We have also found that subjective measures from the patient experience survey had higher ICCs compared to the objective process of care measures. This finding is in agreement with those reported by Campbell et al. [28]. We observed that patient-reported experience had the highest ICC values compared to processes of clinical care and patients’ clinical outcomes, suggesting that there was a higher variation between clinic in this aspect, reflecting potential differences in the approach and level of self-management support provided by each clinic’s healthcare providers. For example, the high ICC value for PACIC item 9 (Given a copy of my treatment plan) suggests that some of the clinics provide their patients with a document or booklet to increase individual awareness on disease control and treatment, whilst the rest of the clinics are less likely to provide such documents.

There are several limitations in this study. The Malaysian healthcare system consists of two sectors: a government-led and funded public sector and a private sector which is largely financed by out-of-pocket payments and private insurance [30]. Results from this study are reflective only of public primary care clinics in Malaysia. Data for processes of care and intermediate outcomes were extracted from medical records. Therefore, the results are more likely affected by quality of documentation and the methods of measurement, which may vary between clinics. Lastly, the estimates for low-prevalence processes of care or patient experience should be interpreted with caution due to greater imprecision when assessing measures that occur infrequently [28].

## Conclusion

We have reported ICC estimates and their respective 95% CIs for a range of measures for T2D and hypertension, which can aid in sample size calculations for cluster randomized controlled trials. This enables more accurate sample size estimation, which allows researchers to calculate realistic recruitment targets whilst reducing the risk of conducting underpowered cluster trials. This is important, particularly in settings where resources are scarce.

## Data Availability

EnPHC-EVA: Facility data were analysed and used for this study. The data are not publicly available due to confidentiality restrictions. However, the data are available from the authors upon reasonable request from the permission of Institute for Clinical Research, Ministry of Health Malaysia. All requests for data should be addressed to the Institute for Clinical Research at contact@crc.gov.my.

## Appendix

These are the R scripts for generating ICC values by using the ICCbin package and the psychometric package for binary variables and continuous variables respectively. The example shown here for binary variables is the proportion of T2D patients with or without hypertension who have their random blood glucose (RBG) measured on a visit day. The example for continuous variables is the mean of HbA1c of T2D patients with or without hypertension.

Codes for binary variables [20]

library (ICCbin)

iccbin (cid = Site_ID, y = RBGonVisitDate, data = dm, method = c(“aov”), ci.type = c(“aov”), alpha = 0.05)

Details

*cid: cluster variable which is Site_ID*

*y: dependent variable*

*data: the name of the dataset*

*method: the method used to compute ICC. In our study, the ANOVA method was used. The code for ANOVA is “aov”*

*ci.type: the method used to compute the confidence intervals. We used “aov”, which was Smith’s large sample approximation*

*alpha: the significance level for CI. Default value is 0.05. This gives the 95% CI*

Codes for continuous variables [22]

library(psychometric)

ICC1.CI(Lab_HbA1cResult, Site_ID, dm, level = 0.95)

Details

*ICC1.CI(dv, iv, data, level = 0.95)*

*ICC1.CI: method used to compute the ICC and CI. ICC1.CI computes ICC from one-way ANOVA, and the CI is computed at the desired level using formulae provided by McGraw and Wong* [31]

*dv: dependent variable*

*iv: cluster variable which is Site_ID*

*data: name of the dataset*

*level: significance level for CI. Default value is 0.95*

## Abbreviations

ACEI: Angiotensin-converting enzyme inhibitor
ANOVA: Analysis of variance
ARB: Angiotensin II receptor blocker
BMI: Body mass index
BP: Blood pressure
CI: Confidence interval
CRCT: Cluster randomized controlled trial
CVD: Cardiovascular disease
DBP: Diastolic blood pressure
DE: Design effect
ECG: Electrocardiography
EnPHC: Enhanced Primary Health Care
EnPHC-EVA: Facility: Evaluation of Enhanced Primary Health Care (EnPHC) interventions in public health clinics
FBG: Fasting blood glucose
HbA1c: Glycated haemoglobin
HDL-C: High-density lipoprotein cholesterol
HMG-CoA: Hydroxymethylglutaryl-coenzyme A
ICC: Intra-cluster correlation coefficient
LDL-C: Low-density lipoprotein cholesterol
PACIC: Patient Assessment of Chronic Illness Care (instrument)
SBP: Systolic blood pressure
SD: Standard deviation
T2D: Type 2 diabetes
TC: Total cholesterol
TG: Triglycerides
