# Correction to: Intra-cluster correlation coefficients in primary care patients with type 2 diabetes and hypertension

**DOI:** 10.1186/s13063-020-04715-2

**Published:** 2020-09-10

**Authors:** Yi Lin Lee, Yvonne Mei Fong Lim, Kian Boon Law, Sheamini Sivasampu

**Affiliations:** 1 Centre for Clinical Trial, Institute for Clinical Research, Ampang Hospital, Ministry of Health, Jalan Mewah Utara, Pandan Mewah, 68000 Ampang, Selangor Malaysia; 2 Centre for Clinical Outcome Research, Institute for Clinical Research, National Institute of Health, Ministry of Health, Kompleks Institut Kesihatan Negara (NIH), No. 1, Jalan Setia Murni U13/52, Seksyen U13, Setia Alam, 40170 Shah Alam, Selangor Malaysia

**Correction to: Trials 21, 530 (2020).**

**https://doi.org/10.1186/s13063-020-04349-4**

Following publication of the original article [1], we were notified of a typographical error in the Method section, page 2, paragraph 4.

The first sentence is incomplete. It should read: “There were four phases of data collection for 31 time points (in months): (1) November 2016 to June 2017 (pre-intervention), (2) August 2017 to June 2019 (post-intervention).” The original article has been corrected.
